# Autologous Transplantation of Adipose-Derived Mesenchymal Stem Cells Markedly Reduced Acute Ischemia-Reperfusion Lung Injury in a Rodent Model

**DOI:** 10.1186/1479-5876-9-118

**Published:** 2011-07-22

**Authors:** Cheuk-Kwan Sun, Chia-Hung Yen, Yu-Chun Lin, Tzu-Hsien Tsai, Li-Teh Chang, Ying-Hsien Kao, Sarah Chua, Morgan Fu, Sheung-Fat Ko, Steve Leu, Hon-Kan Yip

**Affiliations:** 1 Department of Emergency Medicine, E-Da Hospital, I-Shou University, Kaohsiung, Taiwan; 2 Division of General Surgery, Department of Surgery, Kaohsiung Chang Gung Memorial Hospital and Chang Gung University College of Medicine, Kaohsiung, Taiwan; 3 Department of Life Science, National Pingtung University of Science and Technology, Pingtung, Taiwan; 4 Center for Translational Research in Biomedical Sciences, Kaohsiung Chang Gung Memorial Hospital and Chang Gung University College of Medicine, Kaohsiung, Taiwan; 5 Division of Cardiology, Department of Internal Medicine, Kaohsiung Chang Gung Memorial Hospital and Chang Gung University College of Medicine, Kaohsiung, Taiwan; 6 Basic Science, Nursing Department, Meiho University, Pingtung, Taiwan; 7 Department of Medical Research, E-Da Hospital, I-Shou University, Kaohsiung, Taiwan; 8 Department of Radiology, Kaohsiung Chang Gung Memorial Hospital and Chang Gung University College of Medicine, Kaohsiung, Taiwan

## Abstract

**Background:**

This study tested the hypothesis that autologous transplantation of adipose-derived mesenchymal stem cells (ADMSCs) can effectively attenuate acute pulmonary ischemia-reperfusion (IR) injury.

**Methods:**

Adult male Sprague-Dawley (SD) rats (n = 24) were equally randomized into group 1 (sham control), group 2 (IR plus culture medium only), and group 3 (IR plus intravenous transplantation of 1.5 × 10^6 ^autologous ADMSCs at 1h, 6h, and 24h following IR injury). The duration of ischemia was 30 minutes, followed by 72 hours of reperfusion prior to sacrificing the animals. Blood samples were collected and lungs were harvested for analysis.

**Results:**

Blood gas analysis showed that oxygen saturation (%) was remarkably lower, whereas right ventricular systolic pressure was notably higher in group 2 than in group 3 (all p < 0.03). Histological scoring of lung parenchymal damage was notably higher in group 2 than in group 3 (all p < 0.001). Real time-PCR demonstrated remarkably higher expressions of oxidative stress, as well as inflammatory and apoptotic biomarkers in group 2 compared with group 3 (all p < 0.005). Western blot showed that vascular cell adhesion molecule (VCAM)-1, intercellular adhesion molecule (ICAM)-1, oxidative stress, tumor necrosis factor-α and nuclear factor-κB were remarkably higher, whereas NAD(P)H quinone oxidoreductase 1 and heme oxygenase-1 activities were lower in group 2 compared to those in group 3 (all p < 0.004). Immunofluorescent staining demonstrated notably higher number of CD68+ cells, but significantly fewer CD31+ and vWF+ cells in group 2 than in group 3.

**Conclusion:**

ADMSC therapy minimized lung damage after IR injury in a rodent model through suppressing oxidative stress and inflammatory reaction.

## Background

The lung maintains its unique function of effective gaseous exchange because of its remarkably large alveolar surface area, its rich and delicate alveolar capillary network, as well as its physical properties (i.e. elasticity and compliance). On the other hand, it is vulnerable to acute ischemia-reperfusion (IR) injury in situations such as resuscitation from hemorrhagic/septic shock and recovery from cardiac surgeries where pulmonary blood supplies have to be clamped, and also after lung transplantation [1-4]. Inflammatory cells have been reported to be the key coordinators of IR-elicited pulmonary injury in response to inflammatory response and oxidative stress [5-7]. Additionally, the productions of reactive oxygen species (ROS), pro-inflammatory cytokines, and adhesion molecules have also been found to be crucial contributors to lung IR injury [6,8-12].

Acute lung injury of different etiologies is known to be associated with high in-hospital morbidity and mortality [13-15]. Previous studies have shed some light on several potential therapeutic strategies including the use of aprotinin [4], N-acetyl-L-cysteine [16], hypothermia [17], and inhalational nitric oxide [18]. However, the effectiveness of these treatment modalities is still uncertain. A safe and effective therapeutic regimen for patients with acute lung injury, therefore, is eagerly awaited.

Accumulating evidence from studies on animal models and human pulmonary tissue have shown that mesenchymal stem cell (MSC) therapy is of noteworthy potential in improving pulmonary functions in various settings of lung diseases, including acute lung injury [19-23]. In addition to regulating angiogenic [24] and pro-inflammatory [25,26] cytokines associated with MSC treatment, other proposed mechanisms including suppression of inflammatory reaction, immunomodulation, and repair of damaged epithelial cells have also been suggested [19-25]. Interestingly, although the benefits of MSC therapy in improving bleomycin- and endotoxin-induced acute or chronic lung injury using animal models have been extensively investigated [22,24-27], the effect of MSC therapy on IR-induced pulmonary injury in experimental models has seldom been reported [23]. Besides, although bone marrow-derived MSC is the major source of stem cells in these studies [22,24-27], the therapeutic role of adipose-derived mesenchymal stem cells (ADMSCs) in acute IR injury of the lung has not been investigated. Recently, ADMSCs have been reported to have the distinct advantages of being abundant, easy to obtain with minimal invasiveness, and readily cultured to a sufficient number for autologous transplantation without ethical issue of allografting [28]. Moreover, it has been demonstrated that, compared with bone marrow-derived MSCs, ADMSCs secrete significantly more bioactive factors that may account for their superior anti-inflammatory and regeneration-enhancing properties [29]. Since the mechanisms involved in IR injuries of solid organs are complicated including the generation of ROS [30], mitochondrial damage [31,32], and a cascade of inflammatory processes [5-7], similar pathogenesis are supposed to account at least partly for the observed IR injury of the lung. Hence, we hypothesized that administration of ADMSCs has a positive therapeutic impact on pulmonary IR injury at cellular, molecular, and functional levels.

## Methods

### Ethic

All experimental animal procedures were approved by the Institute of Animal Care and Use Committee at Kaohsiung Chang Gung Memorial Hospital (Affidavit of Approval of Animal Use Protocol No. 2008121108) and performed in accordance with the Guide for the Care and Use of Laboratory Animals (NIH publication No. 85-23, National Academy Press, Washington, DC, USA, revised 1996).

### Animal Grouping and Isolation of Adipose-Derived Mesenchymal Stem Cells

Pathogen-free, adult male Sprague-Dawley (SD) rats (n = 24) weighing 300-325 g (Charles River Technology, BioLASCO Taiwan Co., Ltd., Taiwan) were randomized into group 1 (sham control, n = 8), group 2 (IR plus culture medium, n = 8) and group 3 (IR plus autologous ADMSC infusion, n = 8) before isolation of ADMSCs.

The rats in group 3 were anesthetized with inhalational isoflurane 14 days before induction of IR injury. Adipose tissue surrounding the epididymis was carefully dissected, excised and prepared based on our recent report [28]. Then 200-300 μL of sterile saline was added to every 0.5 g of adipose tissue to prevent dehydration. The tissue was cut into < 1 mm^3 ^pieces using a pair of sharp, sterile surgical scissors. Sterile saline (37°C) was added to the homogenized adipose tissue in a ratio of 3:1 (saline: adipose tissue), followed by the addition of stock collagenase solution to a final concentration of 0.5 units/mL. The centrifuge tubes with the contents were placed and secured on a Thermaline shaker and incubated with constant agitation for 60 ± 15 minutes at 37°C. After 40 minutes of incubation, the content was triturated with a 25 mL pipette for 2-3 minutes. The cells obtained were placed back to the rocker for incubation. The contents of the flask were transferred to 50 mL tubes after digestion, followed by centrifugation at 600 g for 5 minutes at room temperature. The fatty layer and saline supernatant from the tube were poured out gently in one smooth motion or removed using vacuum suction. The cell pellet thus obtained was resuspended in 40 mL saline and then centrifuged again at 600 g for 5 minutes at room temperature. After being resuspended again in 5 mL saline, the cell suspension was filtered through a 100 μm filter into a 50 mL conical tube to which 2 mL of saline was added to rinse the remaining cells through the filter. The flow-through was pipetted into a new 50 mL conical tube through a 40 μm filter. The tubes were centrifuged for a third time at 600 g for 5 minutes at room temperature. The cells were resuspended in saline. An aliquot of cell suspension was then removed for cell culture in Dulbecco's modified Eagle's medium (DMEM)-low glucose medium containing 10% FBS for 14 days. Approximately 5.5 × 10^6 ^ADMSCs were obtained from each rat. Flow cytometric analysis was performed for identification of cellular characteristics after cell-labeling with appropriate antibodies on day 0 before cell culture and on day 14 prior to transplantation (Table 1).

**Table 1:** Flow Cytometric Analysis of Adipose-Derived Mesenchymal Stem Cell Surface Markers Prior to (Day1) and Following Cell Culture (Day 14)

Surface markers	Day 1	Day 14	p-value†
CD31+	22.0 ± 3.5	19.3 ± 6.8	0.563
CD34+	14.1 ± 7.8	15.1 ± 14.9	0.844
KDR+	19.7 ± 2.5	17.4 ± 8.2	0.438
C-kit+	3.13 ± 1.80	2.40 ± 1.24	0.563
Sca-1+	3.22 ± 1.49	2.72 ± 2.10	0.688
VEGF+	14.3 ± 5.2	14.7 ± 8.7	1.0
vWF+	15.9 ± 7.6	15.9 ± 7.1	1.0
CD26+‡	18.0 ± 3.7	4.7 ± 4.4	0.031
CD45+¶	14.1 ± 12.5	11.6 ± 12.0	0.844
CD271+	18.4 ± 5.7	16.6 ± 7.6	0.688
CD29+	23.7 ± 8.7	91.4 ± 7.1	0.031
CD90+	35.2 ± 5.8	88.1 ± 10.9	0.031

Data are expressed as %.

n = 6 in each experimental study.

† by Wilcoxon signed rank test for paired data.

‡ Dipeptidyl peptidase IV (DPP-IV)/CD26 indicates a cell surface glycoprotein.

¶ Leukocyte common antigen.

KDR = Kinase insert domain receptor; VEGF = vascular endothelial growth factor; vWF = von Willebrand Factor.

To determine whether culturing ADMSCs had anti-inflammatory and immunomodulatory properties, another 6 rats were used in the current study. The ADMSCs on day 0 prior to and on day 14 after cultivation were utilized for analyzing the mRNA expressions of interleukin (IL)-10, IL-4, adiponectin and interferon-γ using RT-PCR, respectively.

### ADMSC Labeling with CM-Dil, Protocol of IR Induction, and Autologous ADMSC Administration

By day 14 prior to ADMSC infusion, all animals were anesthetized by chloral hydrate (35 mg/kg i.p.) plus inhalational isoflurane and placed in a supine position on a warming pad at 37°C, followed by endotracheal intubation with positive-pressure ventilation (180 mL/min) with room air using a Small Animal Ventilator (SAR-830/A, CWE, Inc., USA). Under sterile conditions, the lung was exposed via a left thoracotomy. Lung IR was then conducted in group 2 and group 3 animals on which a left thoracotomy was performed with the left main bronchus and blood supplies to the left lung totally clamped for 30 minutes using non-traumatic vascular clips before reperfusion for 72 hours. Successful clamping was confirmed by the observation of a lack of inflation of the left lung on mechanical ventilation. Sham-operated rats subjected to left thoracotomy only served as normal controls. The CM-Dil (Vybrant™ Dil cell-labeling solution, Molecular Probes, Inc.) (50 μg/mL) was added to the culture medium 30 minutes before IR procedure for ADMSC labeling. After completion of ADMSC labeling, intravenous infusion of autologous ADMSCs (1.5 × 10^6^) was performed 60 minutes, 6 hours, and 24 hours after reperfusion via the penile vein. The dosage of ADMSCs utilized in the current study was based on our recent reports [33,34]. All animals were sacrificed 72 hours after lung reperfusion after measurement of right ventricular systolic blood pressure (RVSBP). The left lungs were collected for subsequent studies.

### Determination of Oxygen Saturation and Right Ventricular Systolic Blood Pressure (RVSBP)

To determine the effect of ADMSC therapy on arterial oxygen saturation (Sat O_2_), carotid arterial blood gas was analyzed prior to left thoracotomy and at 72 h after the IR procedure. RVSBP, an indicator of pulmonary arterial blood pressure, was assessed at 72 h after the IR procedure prior to sacrificing the animals.

For RVSBP measurement, each animal was endotracheally intubated with positive-pressure ventilation (180 mL/min) with room air using a small animal ventilator. The detailed procedure has been described in our recent report [33]. Briefly, the heart was exposed by left thoracotomy. A sterile 20-gauge, soft-plastic coated needle was inserted into the right ventricle and femoral artery of each rat to measure the RVSBP and systemic arterial pressure, respectively. The pressure signals were first transmitted to pressure transducers (UFI, model 1050, CA, U.S.A.) and then exported to a bridge amplifier (ML866 PowerLab 4/30 Data Acquisition Systems. ADInstruments Pty Ltd., Castle Hill, NSW, Australia) where the signals were amplified and digitized. The data were recorded and later analyzed with the Labchart software (ADInstrument). After hemodynamic measurements, the rats were euthanized with the hearts and lungs harvested. Half of the left lung was fixed in 4% formaldehyde and then embedded in paraffin blocks, while the rest was cut into pieces, frozen in liquid nitrogen and then stored at -80° C until future use.

### Identification of Alveolar Sac Distribution in Lung Parenchyma

Left lung specimens from all animals were fixed in 10% buffered formalin before embedding in paraffin and the tissue was sectioned at 5 μm for light microscopic analysis. After hematoxylin and eosin (H & E) staining, the number of alveolar sacs was determined in a blinded fashion according to our recent study [33]. Three lung sections from each rat were analyzed and three randomly selected high-power fields (HPFs) (100×) were examined in each section. The mean number per HPF for each animal was then determined by summation of all numbers divided by 9.

### Immunofluorescent (IF) Studies and Crowded Score of Lung Parenchyma

IF staining was performed for the examinations of CD68 (macrophage surface marker)+, CD31+, and von Willebrand factor (vWF)+ cells using respective primary antibodies. Irrelevant antibodies were used as controls in the current study.

The extent of crowded area, which was defined as the region of thickened septa in lung parenchyma associated with partial or complete collapse of alveoli on H & E-stained sections, was determined in a blinded fashion. The scoring system adopted was as follows: 0 = no detectable crowded area; 1 = <15% of crowded area; 2 = 15-25% of crowded area; 3 = 25-50% of crowded area; 4 = 50-75% of crowded area; 5 = >75%-100% of crowded area/per high-power field (100 x).

### Western Blot Analysis of Left Lung Specimens

Equal amounts (10-30 mg) of protein extracts from the left lung were loaded and separated by SDS-PAGE using 8-10% acrylamide gradients. Following electrophoresis, the separated proteins were transferred electrophoretically to a polyvinylidene difluoride (PVDF) membrane (Amersham Biosciences). Nonspecific proteins were blocked by incubating the membrane in blocking buffer (5% nonfat dry milk in T-TBS containing 0.05% Tween 20) overnight. The membranes were incubated with monoclonal antibodies against vascular cell adhesion molecule (VCAM)-1 (1: 100, Abcam, Cambridge, MA, USA), intercellular adhesion molecule (ICAM)-1 (1: 2000, Abcam, Cambridge, MA, USA), NAD(P)H quinone oxidoreductase (NQO)-1 (1: 1000, Abcam, Cambridge, MA, USA), connexin43 (Cx43) (1: 2000, Chemicon, Billerica, MA, USA), cytochrom C (Cyt C) (1: 2000, BD, San Jose, CA, USA) and heme oxygense (HO)-1 (1: 250, Abcam, Cambridge, MA, USA), and polyclonal antibodies against TNF-α (1: 1000, Cell Signaling, Danvers, MA, USA) and NFκB (1: 250, Abcam, Cambridge, MA, USA). Signals were detected with horseradish peroxidase (HRP)-conjugated goat anti- mouse, goat anti-rat, or goat anti-rabbit IgG.

The Oxyblot Oxidized Protein Detection Kit was purchased from Chemicon (S7150). The procedure of 2,4-dinitrophenylhydrazine (DNPH) derivatization was carried out on 6 μg of protein for 15 minutes according to manufacturer's instructions. One-dimensional electrophoresis was carried out on 12% SDS/polyacrylamide gel after DNPH derivatization. Proteins were transferred to nitrocellulose membranes which were then incubated in the primary antibody solution (anti-DNP 1: 150) for two hours, followed by incubation with secondary antibody solution (1:300) for one hour at room temperature. The washing procedure was repeated eight times within 40 minutes.

Immunoreactive bands were visualized by enhanced chemiluminescence (ECL; Amersham Biosciences) which was then exposed to Biomax L film (Kodak). For quantification, ECL signals were digitized using Labwork software (UVP). For oxyblot protein analysis, a standard control was loaded on each gel.

### Real-Time Quantitative PCR Analysis

Real-time polymerase chain reaction (RT-PCR) was performed using LightCycler TaqMan Master (Roche, Germany) in a single capillary tube according to the manufacturer's instructions for individual component concentrations. Forward and reverse primers were each designed based on individual exons of the target gene sequence to avoid amplifying genomic DNA.

During PCR, the probe was hybridized to its complementary single-strand DNA sequence within the PCR target. As amplification occurred, the probe was degraded due to the exonuclease activity of Taq DNA polymerase, thereby separating the quencher from reporter dye during extension. During the entire amplification cycle, light emission increased exponentially. A positive result was determined by identifying the threshold cycle value at which reporter dye emission appeared above background.

### Statistical Analysis

Quantitative data are expressed as means ± SD. Statistical analysis was adequately performed by ANOVA followed by Bonferroni multiple-comparison post hoc test. Statistical analysis was performed using SAS statistical software for Windows version 8.2 (SAS institute, Cary, NC). A probability value <0.05 was considered statistically significant.

## Results

### Flow Cytometric Analyses of Adipose-Derived Mesenchymal Stem Cell Surface Markers

Cell surface marker study demonstrated the presence of both endothelial progenitor cells (EPCs) (i.e. CD31+, CD34+, KDR+, Sca-1, C-kit, vWF, VEGF) and MSCs (CD26+, CD29+, CD45+, CD90+, CD271+) prior to and 14 days after cell culturing (Table 1). The percentages of all EPC surface markers were similar between day 0 and day 14 of cell culture. Additionally, the percentages of MSC surface markers of CD45+ and CD271+ cells did not differ between day 0 and day 14 of cell culture. However, compared with day 0, the percentage of cells positive for MSC surface marker CD26 was significantly decreased after 14 days of cell culture. In contrast, the percentages of cells positive for MSC surface markers CD29 and CD90 were substantially increased after cell culture for 14 days. These findings, therefore, indicate that adipocytes from adipose tissue can differentiate into EPCs and ADMSCs in Dulbecco's modified Eagle's medium (DMEM) (containing 10% fetal bovine serum) culture medium. The majority of these cells differentiated into ADMSCs instead of EPCs.

### Arterial Oxygen Saturation and Right Ventricular Systolic Blood Pressure (RVSBP)

**Figure 1:**
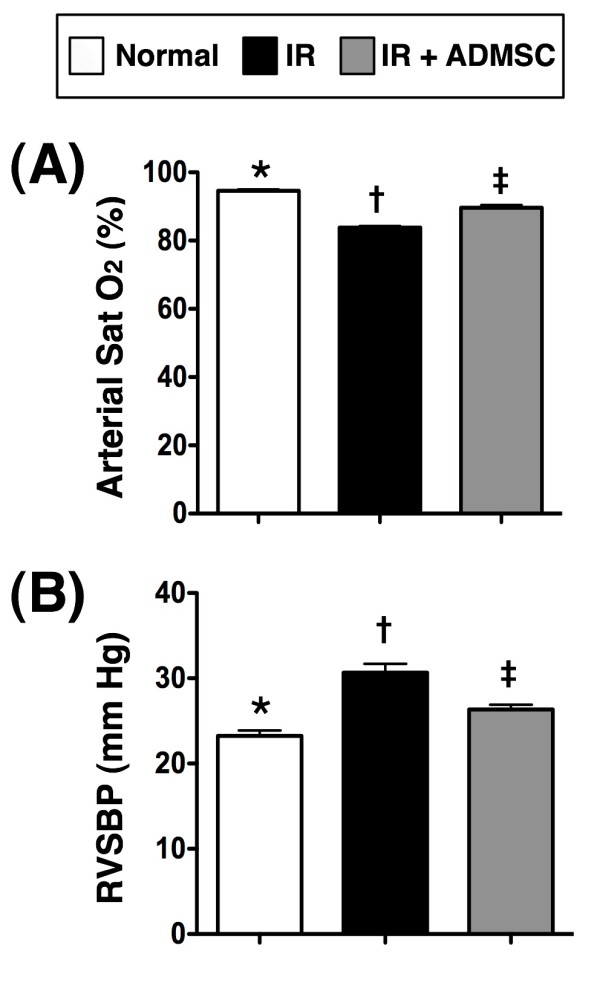
**Arterial Oxygen Saturation and Systolic Blood Pressure in Right Ventricle at 72 Hour after the Procedure**. **(A) **Arterial oxygen saturation (Sat O_2_) at 72 h after ischemia-reperfusion (IR) injury. *p < 0.01 between the indicated groups (n = 8). **(B) **Right ventricular systolic blood pressure (RVSBP). *p < 0.01 between the indicated groups. ADMSC: Adipose-derived mesenchymal stem cells. Symbols (*, †, ‡) indicate significance (at 0.05 level) (by Bonferroni multiple comparison post hoc test).

Sat O_2 _did not differ among control rats (group 1), IR rats (group 2), and IR + ADMSC-treated rats (group 3) prior to the IR procedure (94% vs. 94.3% vs. 93.7%, p > 0.5). However, Sat O_2 _was significantly higher in group 1 than in groups 2 and 3, and notably higher in group 3 than in group 2 at 72 h after the IR procedure (Figure 1A). On the other hand, RVSBP was notably lower in groups 1 and 3 than in group 2, and remarkably higher in group 3 than in group 1 (Figure 1B). These findings indicate that IR injury in the experimental model was successfully created and that ADMSC treatment significantly attenuated IR-elicited lung injury.

### Histopathologic Findings of the Lung

**Figure 2:**
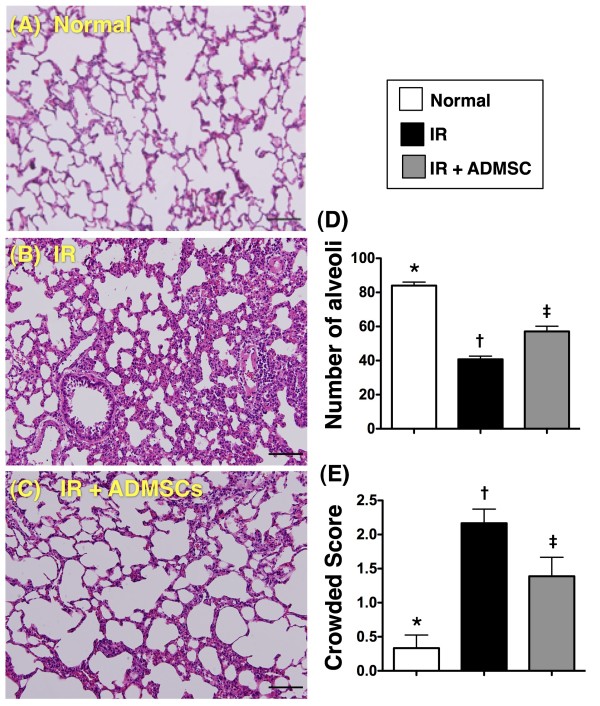
**Impact of Adipose-Derived Mesenchymal Stem Cells (ADMSC) Transplantation on the Severity of IR-Induced Lung Parenchymal Injury. **Number of alveolar sacs and crowded area (was defined in methodology section) under microscope (100×) at 72 h following ischemia-reperfusion (IR) procedure (n = 6). Notably reduced number of alveolar sacs in IR group **(B) **compared with IR + ADMSC **(C) **and normal control **(A) **groups (H & E). Also note more compact lung parenchyma with thickened septum in IR group than in other groups. Septal thickening more prominent in some alveoli in IR group than in IR + ADMSC and normal control groups. Scale bars in right lower corner represent 100 μm. D) *p < 0.001 between the indicated groups. E) *p < 0.0001 between the indicated groups. Symbols (*, †, ‡) indicate significance (at 0.05 level) (by Bonferroni multiple comparison post hoc test).

To evaluate the impact of ADMSC transplantation on the severity of IR-induced lung parenchymal injury, H & E-stained lung sections were examined (Figure 2, A-C). The number of alveolar sacs in left lung was substantially fewer in group 2 than in groups 1 and 3, and notably fewer in group 3 than in group 1 at 72 h after IR (Figure 2D). By contrast, the lung parenchyma was remarkably crowded in group 2 compared with that in groups 1 and 3, and was significantly more crowded in group 3 compared to group 1 (Figure 2E). Additionally, septum thickening was more frequently observed in group 2 than in groups 1 and 3, and this phenomenon was also more frequently present in group 3 than in group 1. These findings, therefore, suggest that ADMSC therapy significantly protected lung parenchyma from IR damage.

### ADMSC Transplantation Attenuated Gene Expression (mRNA) as Related to Vasoconstriction, Inflammation, Oxidative Stress, and Apoptosis in Lung Parenchyma after IR Injury

**Figure 3:**
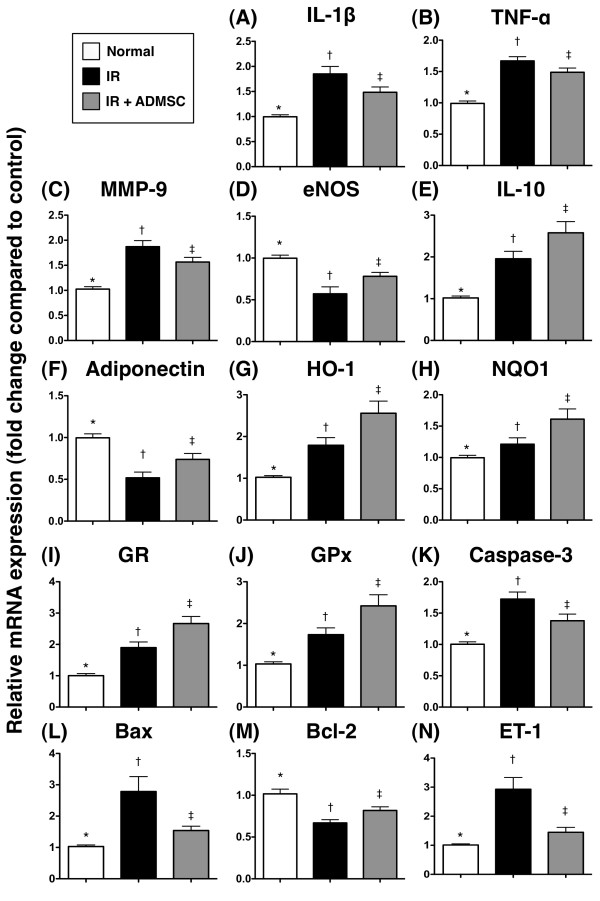
**Analysis of mRNA Expressions of IL-1β, TNF-α, MMP-9, eNOS, IL-10, Adiponectin, HO-1, NQO 1, GR, GPx, Caspase 3, Bax, Bcl-2 and ET-1 in Lung Parenchyma after IR Injury. **Real-time quantitative PCR for gene expression (n = 8). (**A) **Interleukin (IL)-1β mRNA expression. *p < 0.04 between the indicated groups. (**B) **Tumor necrosis factor (TNF)-α mRNA expression. *p < 0.05 between the indicated groups. (**C) **Matrix metalloproteinase (MMP)-9 mRNA expression. *p < 0.04 between the indicated groups. (**D) **Endothelial nitric oxide synthase (eNOS) mRNA expression. *p < 0.04 between the indicated groups. (**E) **IL-10 mRNA expression. *p < 0.05 between the indicated groups. (**F) **Adiponectin mRNA expression. *p < 0.04 between the indicated groups. (**G) **Heme oxygenase (HO)-1 mRNA expression. *p < 0.03 between the indicated groups. (**H) **NAD(P)H quinone oxidoreductase (NQO)-1 mRNA expression. *p < 0.02 between the indicated groups. **(I) **Glutathione reductase (GR) mRNA expression. *p = 0.01 between the indicated groups. (**J) **Glutathione peroxidase (GPx) mRNA expression. *p < 0.03 between the indicated groups. (**K) **Caspase 3 mRNA expression. *p < 0.03 between the indicated groups. **(L) **Bax mRNA expression. *p < 0.03 between the indicated groups. (**M) **Bcl-2 mRNA expression. *p < 0.02 between the indicated groups. (**N) **Endothelin (ET)-1 mRNA expression. *p < 0.03 between the indicated groups. Symbols (*, †, ‡) indicate significance (at 0.05 level) (by Bonferroni multiple comparison post hoc test).

The mRNA expressions of interleukin (IL)-1β, tumor necrosis factor (TNF)-α and matrix metalloproteinase (MMP)-9, three indicators of inflammation, were remarkably higher in group 2 than in groups 1 and 3, and notably higher in group 3 than in group 1 (Figure 3, A-C). Conversely, the mRNA expressions of endothelial nitric oxide synthase (eNOS), IL-10, and adiponectin, the indexes of anti-inflammation, were notably lower in group 2 than in groups 1 and 3, and significantly lower in group 3 than in group 1 (Figure 3, D-F). These findings imply that ADMSC treatment inhibited inflammatory reaction in this experimental setting.

The mRNA expressions of heme oxygenase (HO)-1, NAD(P)H quinone oxidoreductase (NQO) 1, glutathione reductase (GR), and glutathione peroxidase (GPx), four anti-oxidative indicators, were remarkably lower in group 2 than in groups 1 and 3, and notably lower in group 3 than in group 1 (Figure 3, G-J). These findings suggest an induction of anti-oxidative response after IR injury and an enhancement of anti-oxidant effect through ADMSC administration.

The mRNA expressions of caspase 3 and Bax, two pro-apoptotic indexes, were markedly higher in group 2 than those in groups 1 and 3, and notably increased in group 3 compared with those in group 1 (Figure 3, K and 3L). By contrast, the mRNA expression of Bcl-2, an index of anti-apoptosis, was remarkably lower in group 2 than in groups 1 and 3, and significantly reduced in group 3 than in group 1 (Figure 3M). These findings imply that ADMSC treatment exerted anti-apoptotic and mitochondria-protective effects.

The mRNA expression of endothelin (ET)-1, an index of endothelial vasoconstriction and impaired perfusion, was notably higher in group 2 than in groups 1 and 3 and significantly higher in group 3 than in group 1 (Figure 3N). These findings indicate that IR-induced endothelial damage of lung was significantly suppressed after ADMSC treatment.

### Presence of CD31+ von Willebrand Factor (vWF)+ and CD68+ Cells in Lung Parenchyma

**Figure 4:**
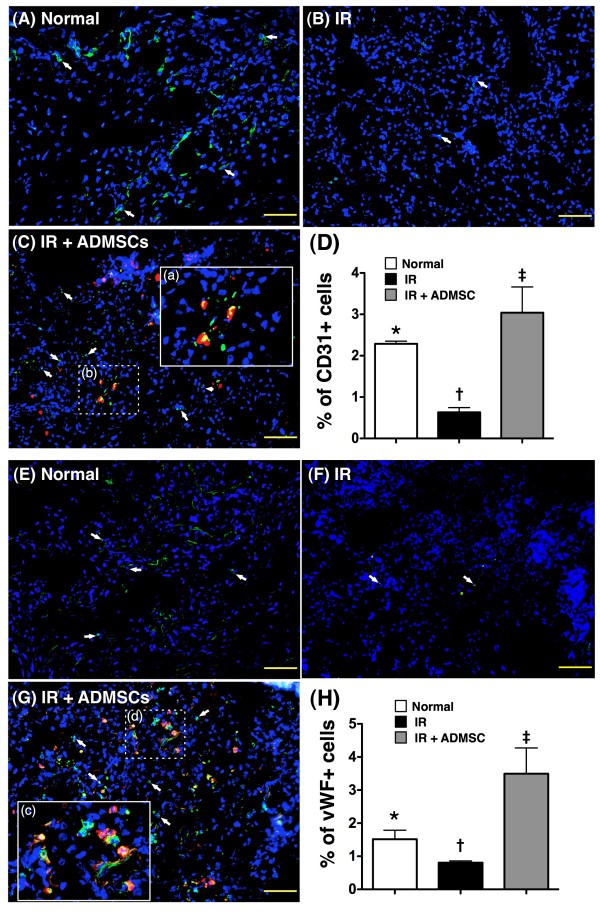
**Presence of CD31+ and von Willebrand Factor (vWF) Cells in Lung Parenchyma**. **(Upper Panel) **Immunofluorescent (IF) staining (200 x) of CD31+ cells with green color in lung parenchyma. Notably fewer number of CD31+ cells (white arrows) in ischemia-reperfusion (IR) group **(B) **than in normal control **(A) **and IR + adipose-derived mesenchymal stem cell (ADMSC) **(C) **groups. **C) **Merged picture from double staining (Dil + CD31) showing mixed color of red and yellow cells [under high magnification (a) of the dotted box (b)], indicating implanted CD31-positive cells presented in lung the lung parenchyma. D) *p < 0.01 between the indicated groups. (**Lower Panel) **IF staining (200 x) of von Willebrand factor (vWF)+ cells with green color in lung parenchyma. Notably reduced number of vWF+ cells (white arrows) in IR group **(F) **than in normal control **(E) **and IR + ADMSC **(G) **groups. **G) **Merged picture from double staining (Dil + CD31) showing mixed color of red, green, and yellow cells [under high magnification (c) of the dotted box (d)], indicating the presence of implanted vWF-positive cells in lung parenchyma. H) *p < 0.001 between the indicated groups. Symbols (*, †, ‡) indicate significance (at 0.05 level) (by Bonferroni multiple comparison post hoc test). Scale bars in right lower corner represent 50 μm. n = 6 in each group.

Fluorescent microscopy revealed expressions of CD31 (Figure 4, A-D) and vWF (Figure 4, E-H), indicators of endothelial cellular phenotypes, in some cells located in lung parenchyma. These findings suggest that angiogenesis occurred in the lung for possible repair of IR injury and tissue regeneration after AMDMSC transplantation.

**Figure 5:**
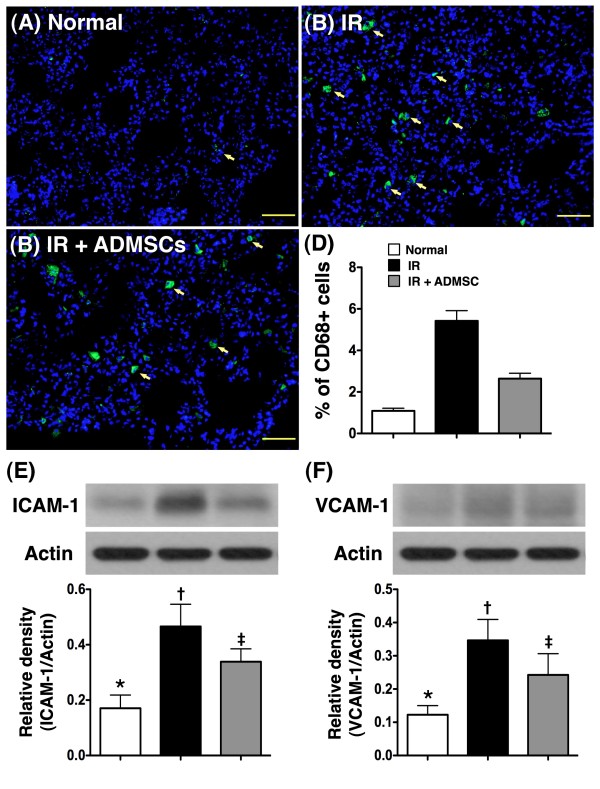
**Adipose-Derived Mesenchymal Stem Cell (ADMSC) Treatment Inhibited Inflammation in Lung Parenchyma after IR Injury**. **(Upper Panel) **Immunofluorescent (IF) staining (200 x) of CD68+ cells (n = 6). Note the notably higher number of CD68+ cells (yellow arrows) in ischemia-reperfusion (IR) group **(B) **than in normal-control **(A) **and IR + ADMSC **(C) **groups. D) *p < 0.001 between the indicated groups. Scale bars in right lower corner represent 50 μm. **(Lower Panel) **Western blot analyses showing significantly higher protein expressions of intercellular adhesion molecule (ICAM)-1 **(E) **and vascular adhesion molecule (VCAM)-1 **(F) **in IR group than in other groups. E) *p < 0.03 between the indicated groups. F) *p < 0.05 between the indicated groups. Symbols (*, †, ‡) indicate significance (at 0.05 level) (by Bonferroni multiple comparison post hoc test).

Immunofluorescent staining demonstrated substantially higher number of CD68+ cells (Figure 5, A-D), a macrophage marker, in group 2 than in groups 1 and 3. The number was also and notably higher in group 3 than in group 1. This finding implies that ADMSC treatment suppressed recruitment of inflammatory cells to pulmonary tissue after IR.

### ADMSC Treatment Inhibited Inflammation and Reactive Oxygen Species Generation in Lung Parenchyma after IR Injury--Assessment at Protein Level

**Figure 6:**
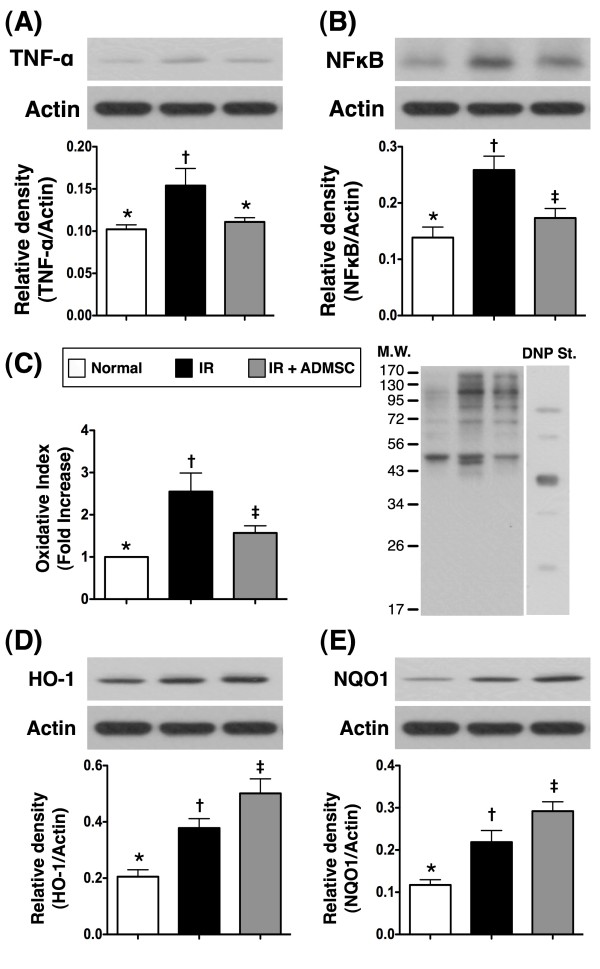
**Adipose-Derived Mesenchymal Stem Cell (ADMSC) Treatment Inhibited Inflammation and Reactive Oxygen Species Generation in Lung Parenchyma after Ischemia-Reperfusion (IR) Injury**. Notably higher protein expressions of TNF-α **(A) **and NF-κB **(B) **in IR group than in normal control and IR + ADMSC groups, but lack of difference between normal control and IR + ADMSC groups. *all p values < 0.04 between the indicated groups. Western blotting **(C) **showing notable increase in the oxidative index, protein carbonyls, in IR group compared with control group and IR + ADMSC group, and notably higher in IR + ADMSC group than in control group. *p < 0.05 between the indicated groups. Remarkably higher protein expressions of HO-1 **(D) **and NQO-1 **(E) **in IR and IR + ADMSC groups than in control group, and markedly higher in IR + ADMSC group than in IR group. *all p values < 0.04 between the indicated groups. n = 6 for each group. Symbols (*, †, ‡) indicate significance (at 0.05 level) (by Bonferroni multiple comparison post hoc test).

Western blot analyses demonstrated notably higher protein expressions of VCAM-1, ICAM-1 (Figure 5, E and 5F), TNF-α, and NF-κB (Figure 6, A and 6B), four acute inflammatory biomarkers, in group 2 than those in groups 1 and 3, and in group 3 compared with those in group 1 following acute lung IR injury. In addition, the protein expression of oxidative stress (Figure 6C), an indicator of ROS activity, was increased several folds in group 2 compared with that in groups 1 and 3, and significant higher in group 3 than that in group 1. In contrast, the protein expressions of HO-1 and NQO-1 (Figure 6, D and 6E), two anti-oxidative biomarkers, were remarkably higher in group 3 than those in groups 1 and 2, and significantly higher in group 2 than those in group 1. These findings further suggest that ADMSC treatment contributed to the anti-inflammatory and anti-oxidative effects after IR-induced pulmonary injury in this study.

**Figure 7:**
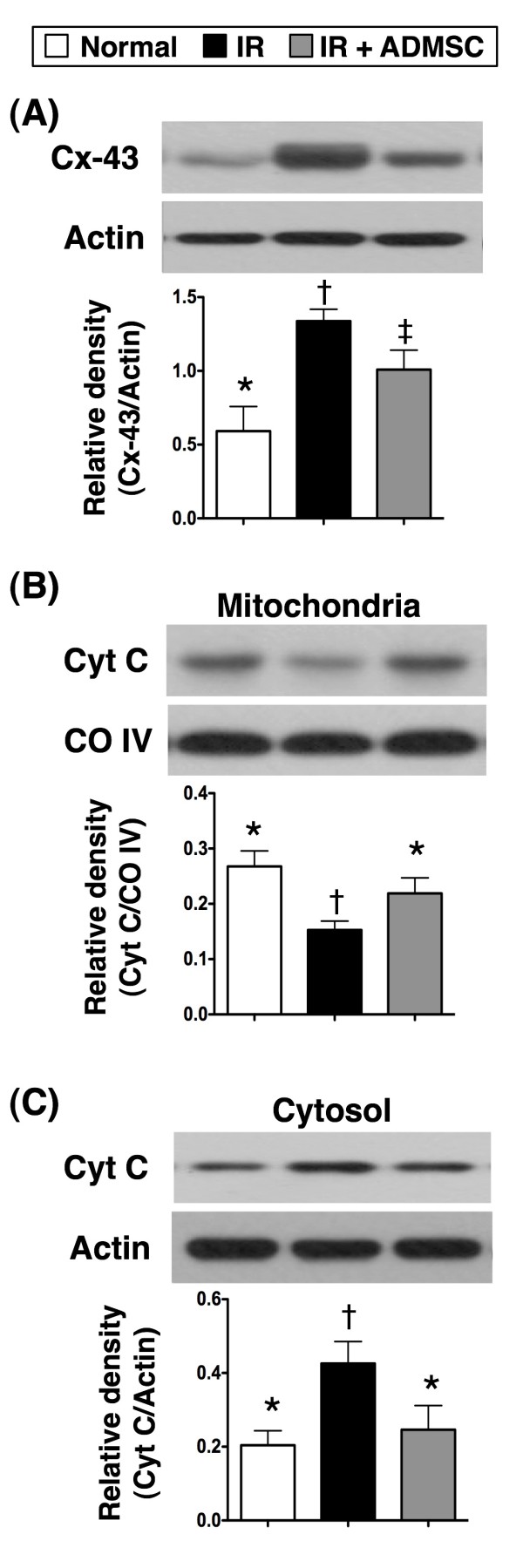
**Adipose-Derived Mesenchymal Stem Cell (ADMSC) Treatment Inhibited apoptosis in Lung Parenchyma after Ischemia-Reperfusion (IR) Injury**. Notably elevated protein expression of connexin (Cx)43 in IR group than in normal control and IR + ADMSC group, and higher in IR + ADMSC group than in normal control group. *p < 0.04 between the indicated groups. **B) **Notably suppressed mitochondrial protein expression of cytochrome c in IR group than in normal-control and IR + ADMSC group, and higher in IR + ADMSC group than in normal control group. *p < 0.03 between the indicated groups. **C) **Remarkably enhanced cytosolic protein expression of cytochrome C (Cyt C) in IR group than in normal control and IR + ADMSC group, and higher in IR + ADMSC group than in normal control group. *p < 0.03 between the indicated groups. Symbols (*, †, ‡) indicate significance (at 0.05 level) (by Bonferroni multiple comparison post hoc test). n = 6 for each group. COIV=cytochrome oxidase subunit IV.

Protein overexpression of Cx43 (Figure 7A), an index of smooth muscle proliferation after an acute injury, was remarkably higher in group 2 than that in groups 1 and 3, and significantly higher in group 3 than that in group 1. Besides, mitochondrial cytochrome c (Figure 7B), an index of mitochondrial integrity, was notably reduced in group 2 compared with that in groups 1 and 3, but it did not differ between group 1 and group 3. On the other hand, an increase of cytochrome c in cytosol (Figure 7C), an index of mitochondrial damage, was notably higher in group 2 than that in groups 1 and 3. However, no significant difference was noted between group1 and group 3. These findings further suggest that ADMSC therapy protected lung parenchyma from IR damage, possibly through suppression of smooth muscle proliferative response and preservation of mitochondrial integrity.

## Discussion

This study, which utilized a rodent model to investigate the therapeutic impact of ADMSC treatment on IR-elicited acute lung injury, provided several striking implications. First, not only did ADMSC treatment significantly preserve architectural integrity of lung parenchyma, but it also remarkably reduced the deterioration of pulmonary function after IR injury. Second, ADMSC therapy significantly ameliorated IR-induced pulmonary artery hypertension. Third, ADMSC treatment was associated with early-onset anti-inflammatory, anti-oxidative, and pro-angiogenic effects in pulmonary tissue after IR injury.

### ADMSC Transplantation Ameliorates Inflammation and Oxidative Stress, and Attenuates Apoptosis and Architectural Damage in Lung Following Acute IR Injury -- Role of Immune Modulation

Undoubtedly, the lung is vulnerable to damage through a variety of etiologies because of its distinctive anatomical feature, circulation, and its unique function in gaseous exchange [1-4]. Besides, similar to the central nervous system and myocardium, the lung has only minimal ability of regeneration after injuries. In addition, ROS production, immune response, and inflammatory reaction elicited by a primary insult are usually rigorous and cause irreversible secondary damage to the lung parenchyma [5-15]. The appropriate treatment strategy toward acute lung injury, therefore, is a formidable challenge to physicians.

Experimental studies have recently shown that therapy with bone marrow-derived MSCs markedly attenuated endotoxin- or belomycin-induced lung injury through suppressing the generation of pro-inflammatory cytokines and inflammatory reaction [19-23,25,26]. One important finding in the current study is that the mRNA expressions of IL-1β, TNF-α, and MMP-9 as well as the protein expressions of ICAM-1, VCAM-1, TNF-α, NF-κB, and oxidative stress were remarkably increased in group 2 compared to those in normal controls after acute IR injury. Moreover, immunofluorescent staining identified substantially higher number of infiltrated CD68+ cells (inflammatory cells of macrophages) in injured lung parenchyma in IR group than in normal control. Our findings, therefore, reinforce those of previous studies [5-15]. Of particular importance is that, as compared with IR-injured animals without treatment, the expressions of these inflammatory and oxidative biomarkers at gene, cellular, and protein levels were markedly suppressed in animals following ADMSC treatment. In this way, our findings corroborate those of other recent studies [19-23,25,26].

There are several principal findings in the current study. RT-PCR and Western blot analysis demonstrated remarkably lower expressions of NQO-1 and HO-1, the scavengers for free radicals, in group 2 as compared with group 3 after ADMSC treatment. Besides, RT-PCR revealed significantly lower expressions of anti-oxidative enzymes GR and GPx in group 2 after IR injury compared to those in group 3 following ADMSC administration. In addition, significantly reduced mRNA expressions of Bax and caspase 3 and notably enhanced mRNA expression of Bcl-2 were demonstrated in IR-injured animals with ADMSC treatment compared with those without. Importantly, histological, hemodynamic, and blood gas analyses showed, respectively, that lung parenchymal damage, elevated pulmonary arterial blood pressure, and impaired gaseous exchange were substantially improved in group 3 following ADMSC administration. In other words, these findings suggest that ADMSC treatment preserved lung function, at least in part, through inhibiting inflammatory reactions and suppressing oxidative stress and apoptosis in the experimental setting of acute lung IR injury. Consistently, one recent report has also shown that MSC therapy prevented IR injury of lung and improved pulmonary function through inhibiting cellular apoptosis and generation of inflammatory mediators [23].

Growing evidence has shown that MSCs have distinct immunomodulatory property that participates in down-regulation of inflammatory reaction and cellular apoptosis under ischemic condition [28,35,36]. Interestingly, the present study demonstrated notably increased pulmonary mRNA expressions of IL-10 and adiponectin in animals with ADMSC therapy compared with those without. In concert with the finding of the present study, one previous study has also shown that MSC therapy attenuated endotoxin-induced acute lung injury through up-regulation of anti-inflammatory cytokine IL-10 [25]. Accordingly, in addition to reinforcing the findings of previous studies [17,25,28,32], the results of the current study suggest that ADMSC treatment also preserved pulmonary function through immunomodulation in this experimental setting.

### Transplantation of ADMSCs Initiates Angiogenesis -- An Ischemia-Relieving Phenomenon

Studies have recently revealed that angiogenesis/vasculogenesis is one of the key mechanisms accounting for the improvement in ischemic organ dysfunction after stem cell therapy [28,35,37,38]. The results of the present study showed that cells positively stained for endothelial markers (i.e. CD31 and vWF) were abundantly present in alveolar septum and lung parenchyma in animals having receiving ADMSC treatment. Furthermore, mRNA expression of eNOS, an indicator of angiogenesis, was remarkably increased, whereas the expression of ET-1, an indicator of endothelial vasoconstriction and impaired perfusion, was notably suppressed in animals with ADMSC treatment compared with those without. Taken together, our findings, in addition to corroborating those of previous studies [28,35,37,38], suggest that ADMSC treatment may, at least in part, protect lung parenchyma and preserve lung function after IR injury through enhancing angiogenesis and relieving ischemia.

### ADMSC Treatment Alleviates Connexin43 Protein Over-Expression after Acute Lung Injury

**Figure 8:**
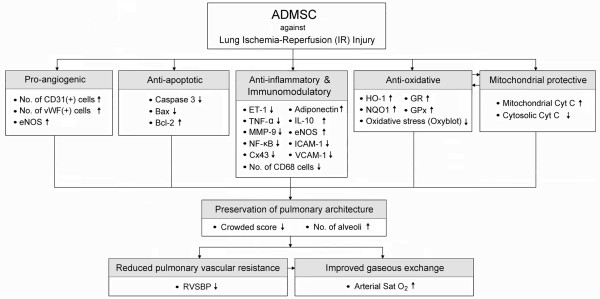
**Proposed mechanisms underlying the improvement in pulmonary functions after adipose-derived mesenchymal stem cell (ADMSC) treatment in a rodent model of pulmonary ischemia-reperfusion (IR) injury**. vWF :von Willebrand factor, ET-1: Endothelin-1; TNF-α: Tumor necrosis factor-α; MMP-9: Matrix metalloproteinase-9; NF-κB: Nuclear factor kappa B; Cx43: Connexin43; IL-10: Interleukin-10; eNOS: Endothelial nitric oxide synthase; ICAM-1: Intercellular Adhesion Molecule-1; VCAM-1: Vascular cell adhesion molecule; HO-1: Heme oxygenase-1; NQO1: NAD(P)H quinone oxidoreductase; GR: Glutathione reductase; GPx: Glutathione peroxidase; Cyt C: Cytochrome C; RVSBP: Right ventricular systolic blood pressure; Sat O_2_: Oxygen saturation.

Recent study has shown an association between Cx43 protein over-expression and smooth muscle cell/fibroblast proliferation in acute and early phase of lung injury [39]. Undoubtedly, increased septal thickness resulted from smooth muscle cell and fibroblast proliferation as well as fibrosis of lung parenchyma imposes a barrier to effective gaseous exchange that could, in turn, cause hypoxemia. One of the principal findings of the current study is the remarkable increase in Cx43 protein expression, an index of smooth muscle cell proliferation after acute lung injury, in animals after acute lung IR. Additionally, the number of alveolar sacs was significantly decreased, whereas the crowded score of lung parenchyma was substantially increased in the animals after pulmonary IR injury. Importantly, these pathological findings and hypoxemia phenomenon were markedly attenuated after ADMSC therapy. These findings, in addition to supporting those of a recent study [39], may also suggest that ADMSC therapy attenuates acute IR lung injury through inhibiting smooth muscle cell proliferation and fibrosis in lung parenchyma. The proposed mechanisms according to the results of the current study have been summarized in Figure 8.

## Study Limitations

This study has limitations. First, since the current study was only designed to investigate the therapeutic potential of ADMSC in an experimental model of acute IR lung injury, it did not provide insight into the potential long-term outcome of ADMSC treatment in this experimental setting. Second, the dosage of ADMSC utilized in the present study was based on our recent reports [33,34]. No experiment, however, was performed to elucidate the best dosage of ADMSC in this particular experimental setting. Third, although the proposed mechanism may serve as a scaffold outlining the possible relationships among our study parameters, the exact mechanisms underlying the observed improvement in IR-induced pulmonary injury through ADMSC administration are likely to be more complex and possibly involve multiple compensatory routes. The relative importance of one specific pathway, however, was not investigated in the present study. Solid cause-and-effect relationships underlying the exact mechanisms, therefore, remain to be elucidated.

## Conclusion

ADMSC treatment remarkably attenuated lung parenchymal injury and improved lung function after acute IR injury. The key mechanisms underlying the positive therapeutic impact could be due to suppression of inflammatory response and oxidative stress as well as enhancement of angiogenesis.

## Competing interests

The authors declare that they have no competing interests.

## Authors' contributions

CKS and CHY participated in the design of the study, data acquisition and analysis as well as drafting the manuscript. LTC, YHK, and YCL were responsible for the laboratory assay and troubleshooting. SC, THT, MF, and SFK participated in data acquisition, analysis, and interpretation. SL and HKY conceived of the study, and participated in its design and coordination and helped to draft the manuscript. All authors read and approved the final manuscript.
